# Experimental development of the epigenomic library construction method to elucidate the epigenetic diversity and causal relationship between epigenome and transcriptome at a single-cell level

**DOI:** 10.5808/gi.21078

**Published:** 2022-03-31

**Authors:** Kyunghyuk Park, Min Chul Jeon, Bokyung Kim, Bukyoung Cha, Jong-Il Kim

**Affiliations:** 1 Genomic Medicine Institute, Medical Research Center, Seoul National University, Seoul 03080, Korea; 2 Department of Obstetrics and Gynecology, Seoul National University Hospital, Seoul 03080, Korea; 3 Department of Biomedical Sciences, Seoul National University College of Medicine, Seoul 03080, Korea; 4 Department of Biochemistry and Molecular Biology, Seoul National University College of Medicine, Seoul 03080, Korea

**Keywords:** cellular heterogeneity, chromatin accessibility, DNA methylation, histone post-translational modifications (PTMs), single-cell epigenome, single-cell multiome

## Abstract

The method of single-cell RNA sequencing has been rapidly developed, and numerous experiments have been conducted over the past decade. Their results allow us to recognize various subpopulations and rare cell states in tissues, tumors, and immune systems that are previously unidentified, and guide us to understand fundamental biological processes that determine cell identity based on single-cell gene expression profiles. However, it is still challenging to understand the principle of comprehensive gene regulation that determines the cell fate only with transcriptome, a consequential output of the gene expression program. To elucidate the mechanisms related to the origin and maintenance of comprehensive single-cell transcriptome, we require a corresponding single-cell epigenome, which is a differentiated information of each cell with an identical genome. This review deals with the current development of single-cell epigenomic library construction methods, including multi-omics tools with crucial factors and additional requirements in the future focusing on DNA methylation, chromatin accessibility, and histone post-translational modifications. The study of cellular differentiation and the disease occurrence at a single-cell level has taken the first step with single-cell transcriptome and is now taking the next step with single-cell epigenome.

## Introduction

**Fig. 1.**
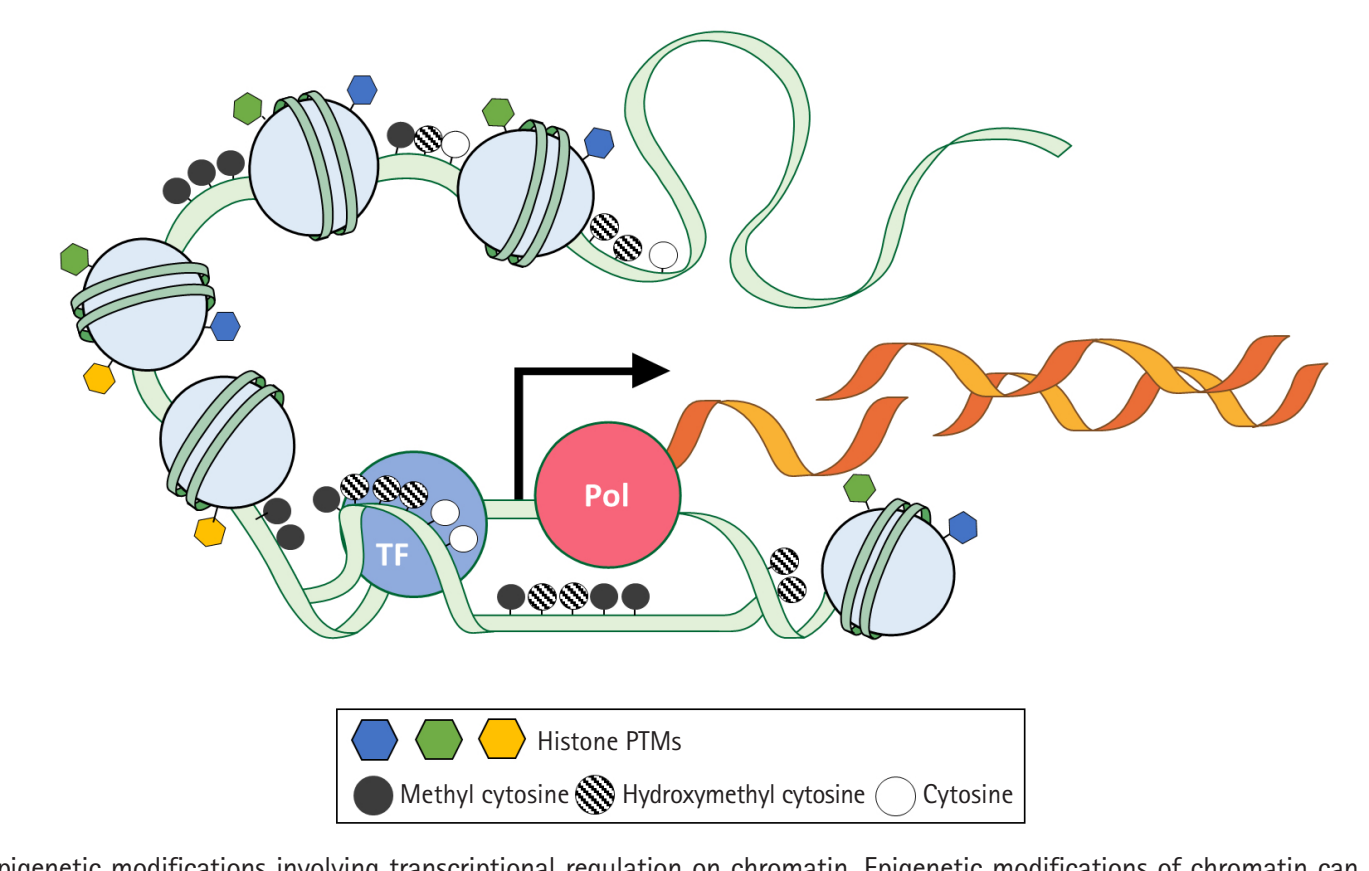
Epigenetic modifications involving transcriptional regulation on chromatin. Epigenetic modifications of chromatin can influence gene expression. Diverse cellular landscapes of nucleosome density, DNA methylation, and histone modifications on identical genomes allow cellular heterogeneity by specifying different gene expression profiles. The light green line is DNA, orange lines are transcripts, and light blue circles are nucleosomes. The blue circle is a transcription factor (TF), and the pink circle is polymerase (Pol). PTM, post-translational modification.

Multicellular organisms constitute multiple types of tissues with identical or closely identical genomes. Those multiple tissues are originated and differentiated from the zygote with a systemic gene expression program of each tissue, which is comprised of multiple types of cells. Current single-cell transcriptome study provides high resolution of the transcriptome map in a single tissue [1-3]. Previously unidentified subpopulation and rare-population of cells are observed with their gene expression profiles at a single-cell level. However, the study of single-cell transcriptome has a limitation to understand the principle and causality of comprehensive transcriptomic regulation on the chromatin, which is a complex of DNA and protein found in all eukaryotic cells [4]. ‘How are cell or tissue-specific expression patterns or framework specified and maintained with the same genome?’ and ‘How does cell or tissue retain the information of external signal even after no more signal exists over several divisions?’ The epigenetic field has introduced and focused on answering these questions. Particularly, epigenetic modifications of chromatin that include nucleosome density, DNA methylation, and histone modifications on identical genome give cells a higher cellular heterogeneity and specificity in a single tissue or single organism by the regulation of gene expression with their inheritable and reversible characteristics during cellular maintenance and differentiation (Fig. 1). In recent years, single-cell methods have been actively applied to the study of epigenetics and explain the causal correlation and maintenance of transcriptome at a single-cell level [5-7]. Finally, epigenetic analysis meets a suitable method. The single-cell epigenomics let us study cellular differentiation, including development, cellular heterogeneity among morphologically same cells, and disease progression with microenvironment deeper than the previous. Although single cell‒specific information on epigenetic features had been notably demanded to study cell identity, it has been challenging to observe them at a single-cell level due to the absence of appropriate techniques and methods. With the rapid development of single-cell technology and methods in recent years, it has become possible to study epigenome at the single-cell level and understand associated transcriptome. Previous effort to make epigenetic encyclopedia [8,9] is now expanding to the single-cell method. The single-cell epigenetic encyclopedia is an ideal path for studying the characteristics of cellular heterogeneity [6,7,10].

## Core Techniques and Methods for Single-Cell Epigenomic Library Construction

**Fig. 2.**
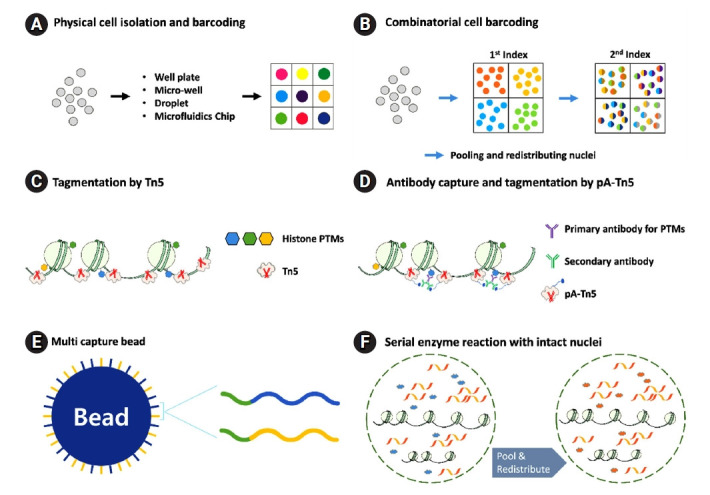
Core techniques and methods of single-cell epigenomic library construction. Single-cell epigenomic sequencing requires more diverse techniques and methods than single-cell RNA sequencing. For single-cell barcoding, physical cell isolation and barcoding (A) and combinatorial cell barcoding (B) are required. Tagmentation by Tn5 (C) improves genomic library construction by performing simultaneous fragmentation and tagmentation, performed separately in the past. Antibody-capturing protein-A fusion Tn5 (D) recognizes specific sites and performs tagmentation simultaneously. Multi-capture beads (E) and the strategy of a serial enzyme reaction with intact nuclei (F) enable multiome library construction for droplet-based and sci-seq methods, respectively. PTM, post-translational modification.

Single-cell epigenomic library construction requires more diverse techniques and methods in addition to single-cell RNA sequencing (Fig. 2). In a large category, physical cell isolation and barcoding (Fig. 2A) and combinatorial cell barcoding (Fig. 2B) are used to label single cells, which are almost the same as RNA sequencing. Tagmentation by Tn5 transposase (Fig. 2C) improves genomic library construction by performing simultaneous fragmentation and tagmentation, performed separately in the past [11,12]. Tagmentation by Tn5 transposase is also the base for the chromatin accessibility assay, which leverages the preferred binding feature of Tn5 to open chromatin. In recent years, going further technically, the method using antibody-capturing protein-A fusion Tn5 (pA-Tn5) (Fig. 2D) has been emerged. The pA-Tn5 recognizes specific sites and performs tagmentation simultaneously [13], which enables us to perform a single-cell epigenomic study of histone post-translational modifications (PTMs) and implies the expansion of the method to any protein that binds chromatin. For a multimodal library construction, multi capture bead (Fig. 2E) and serial enzyme reaction (Fig. 2F) are essential techniques for droplet-based and sci-seq method (single-cell combinatorial indexing sequencing [3]), respectively. Dual or triple capture beads can have more than two kinds of different capture-seq so that the bead can acquire multimodal cellular information, and the strategy of performing two different enzyme reactions serially (Fig. 2F) while pooling and redistributing of nuclei in the middle allows the library to have combinatorial indexes and multimodal information at the same time. Every single-cell epigenomic library construction method combines these core techniques and methods (Tables 1 and 2) [14-33]. We will examine the most recent development in single-cell epigenomics in each epigenetic modification (DNA methylation, chromatin accessibility, histone PTMs, multiome), including the multimodal method.

## DNA Methylation, a Representative Marker for Cell Identity: Hardness and Solution of Single-Cell Library Construction Method Development

Most animals have a comprehensive system of DNA methylation that involves the establishment, removal, maintenance, and recognition of methyl-cytosine [34,35]. Furthermore, DNA methylation is globally reprogramed during gamete development and embryogenesis [36,37] and is highly correlated with cellular identity, including pluripotency, age, and various diseases, particularly cancers [38-45]. Therefore, the study of single-cell methylome had been extensively demanded to observe different states of the methylome. Previous bulk sequencing methods of DNA methylation had already demonstrated cellular heterogeneity of DNA methylation. The percent level of DNA methylation per site rarely appears in 0 or 100. This infers that the methylation of the same DNA region from various cells in a single methylome can have a different methylation state. Furthermore, a comparison of tissue-specific methylome showed tissue specificity of DNA methylation [9,46-49]. These results strongly imply cellular heterogeneity of methylome. Therefore, developing the method for single-cell methylome construction is highly required. However, there are two practical obstacles. The first obstacle is the harsh chemical treatment of DNA. The gold standard of DNA methylation library construction requires chemical preprocessing of the genomic DNA, known as a bisulfite treatment, which converts cytosine to uracil by hydrolytic deamination. At the same time, methyl-cytosine remains unaffected [50]. In the following steps, uracil is amplified and sequenced as thymine. Therefore, the bisulfite library allows the discrimination of methyl-cytosine from unmethylated cytosine at a single base resolution. However, bisulfite treatment for sufficient cytosine to thymine conversion results in DNA loss, fragmentation, and biased sequencing data simultaneously [51-53]. Consequently, due to the DNA loss, whole genome amplification for single-cell methylome construction is required after bisulfite treatment [54-57]. Recently, the bisulfite-free method utilizing methylation-sensitive restriction enzyme has been developed for single-cell methylome (epigenomics and genomics of single cells analyzed by restriction [16], epigenomics, and genomics of single cells analyzed by restriction). However, the region of analysis is restricted to enzyme recognition sites. The second practical obstacle is the cost of sequencing that increases significantly in proportion as the number of cells increases. Since DNA methylation is observed in most of the genome, methylome analysis targets the whole genome, unlike the transcriptome analysis, which only targets mRNA sequences. Although the single-cell reduced-representation bisulfite sequencing (scRRBS) method [15] is optimal to overcome this obstacle, it does not efficiently examine a large number of critical regulatory elements in mammalian genomes. A recent method of extended-representation bisulfite sequencing (XRBS) is performed at a single-cell level with enriching informative methylation profile in promoters, enhancers and, CTCF binding sites [17]. In terms of single-cell methylome data, embryonic stem cells displayed cellular heterogeneity of DNA methylation [54] and the single-cell methylome with combinatorial cellular barcoding discriminated cellular identity by methylome [14]. The epi-gSCAR also showed cellular heterogeneity of DNA methylation by obtaining 506,063 CpG methylation variants from single acute myeloid leukemia-derived cells [16]. Single-cell XRBS has also sampled leukemia cells and featured methylation variability across individual cells and the highest cell-to-cell methylation variability in heterochromatic regions with the tri-methylation mark at the lysine residue of histone 3 (H3K9me3) [17]. All studies of single-cell DNA methylome show apparent cellular heterogeneity. Although none of the single-cell methylome method has resolved both obstacles of sample loss from harsh chemical treatment and high cost as of now, we can still collect single-cell methylome data with the current method (Table 1). Therefore, a data accumulation for understanding the meaning of DNA methylation heterogeneity and the development of the method should be considered together.

## Chromatin Accessibility Providing Binding Sites for Transcription Factors: Single-Cell Library Construction Methods and Their Research Outputs

Nucleosomes comprising histones are found in the nuclei of all eukaryotic cells. Interestingly, chromatin structure with nucleosome shall consist of two distinct structural states: the first one is heterochromatin, which is highly compacted and less accessible to DNA binding proteins than other chromatin regions, and the other one is euchromatin, which is loosely packed and less intense than heterochromatin. The chromatin accessibility of cis-regulatory regions, such as enhancers and promoters around the transcription start site, is crucial to gene regulation, which regulates the binding of various proteins and interacts with other epigenetic markers, including DNA methylation, histone modifications, and non-coding RNA. Two methods of DNase I hypersensitive site sequencing (DNase-seq) [58] and assay for transposase-accessible chromatin sequencing (ATAC-seq) (Fig. 2) [59] have been performed for numerous studies with bulk samples over the past decade and have now been extending to single-cell experiments. Early studies of one single-cell DNase-seq (scDNase-seq) and two of the single-cell ATAC-seq (scATAC-seq) [18,19,60] used physical compartmentalization into each well, combinatorial cellular indexing, and microfluidics for barcoding single cells, respectively (Fig. 2). They clearly showed the cellular variation of chromatin accessibility. Recent droplet-based scATAC-seq can examine tens of thousands of single cells at one experiment [61]. This study discovered new cell types and regulatory elements of the adult mouse brain. It demonstrated cis- and trans-regulatory landscape changes across cell types between resting and stimulated human bone marrow. The study of single-cell chromatin accessibility features considerable cellular variation within a tissue. MNase-seq for nucleosome positioning directly measures nucleosome-bound regions, in contrast to the DNase and ATAC-seq, which measure nucleosome-free regions [62]. This method has also been adapted for single-cell analysis recently [20]. Two features of nucleosome positioning are shown in this study. First, heterochromatin positions or regions show considerable variation across different cells but are highly uniformly spaced. Second, nucleosome positioning at the transcription start site of active genes shows slight variation across other cells, but they are heterogeneously distributed compared to heterochromatic regions [20]. The single-cell library construction method for chromatin accessibility has been developed at a relatively faster rate than single-cell omics for DNA methylation and histone modifications, owing to the development of the tagmentation method (Fig. 2C and 2D) [12]. The tagmentation method using Tn5 transposase performs DNA fragmentation and adaptor tagging simultaneously and has also become a foundation for the single-cell omics of histone modifications.

## Histone PTMs, Markers for the Active and Repressive Transcriptional Status of the Genes: The Latest Development of the Single-Cell Library Construction Methods and Their Research Outputs

The nucleosome consists of 147 base pairs of DNA wound around histone octamers, which is a fundamental subunit of chromatin inside the nucleus. Two copies of each histone protein H2A, H2B, H3, and H4 compose a single nucleosome. Numerous studies have confirmed that chemical modifications of the amino-terminal tails of histone proteins influence transcription and show a correlation with chromatin accessibility. This regulation is also involved in a complex interplay with DNA methylation [63]. DNA methylation generally shows a higher correlation with various H3 methylation states than the DNA sequence [64]. Each histone modification displayed distinct interactions with DNA methylation. Among multiple modifications, methylation of lysine 4, 9, and 27 of H3 (H3K4me, H3K9me, H3K27me) and acetylation of lysine 27 of H3 (H3K27ac) are extensively studied due to their strong correlation with transcriptional states and inheritable characteristics during cell division. Tri-methylation of H3K4 (H3K4me3) is a hallmark of a transcriptionally permissive state enriched in promoter regions of active genes. In contrast, tri-methylation of H3K9 (H3K9me3) and H3K27 (H3K27me3) are representative repressive histone PTMs. H3K27 acetylation (H3K27ac) is an active enhancer mark enriched in the transcription start site's proximal and distal regions. The method of chromatin immunoprecipitation followed by sequencing (ChIP-seq) had been the gold standard to study extensive PTMs-DNA interactions [65-68] with the bulk sample. This method has been expanded to single-cell omics to explore cellular heterogeneity of histone PTMs of a mixture of mouse embryonic stem cells, fibroblasts, and hematopoietic progenitors [21]. This study applied drop fluidics to label each DNA sequence of single cells at the beginning of the protocol. Then those single cells were immunoprecipitated with specific antibodies in the presence of cell barcode. Recently, preprocessing of cells with tagmentation containing antibody reaction was introduced. Various methods that do not rely on immunoprecipitation procedure by using fused MNase with protein A (single-cell chromatin immunocleavage sequencing [scChIC-seq] [22]) or fused Tn5 transposome with protein A (index multiplexing antibody-guided chromatin tagmentation sequencing [23], single-cell chromatin integration labelling sequencing [24], combinatorial barcoding and targeted chromatin release [CoBATCH] [25], single-cell cleavage under targets and tagmentation (scCUT&Tag) [26,69]) improve DNA fragment recovery and reads per cell. Notably, the scCUT&Tag method was used for histone PTMs and transcription factors at the single-cell level [69]. Likewise, CoBATCH method was also used for polymerase alongside histone PTMs [25]. Analysis of single-cell regulatory elements, including binding sites of transcription factors and polymerase with histone PTMs, further enhances our understanding of transcriptional regulation regarding cellular heterogeneity. Furthermore, those methods can be adapted for any DNA and chromatin binding proteins at a single-cell level. Notably, DNA methylation analysis can also be done with those methods by utilizing antibody for methyl-cytosine. The entire study demonstrated above observed cellular heterogeneity of histone PTMs of targeted tissues. For a concrete example, the recent scCUT&Tag profile sufficiently determined cell identity by histone PTMs and showed the regulatory feature of promoter bivalency of active (H3K4me3) and repressive (H3K27me3) marks, spreading of H3K4me3 and promoter-enhancer connectivity of the mouse central nervous system [69].

## Multiome, Single-Cell Epigenome Library with Transcriptome: Current Development of the Methods and Their New Research Findings with a Future Direction

Numerous studies have demonstrated that gene expression is maintained and changed based on epigenetic information [7,63,70]. Therefore, multi-omics library construction of parallel epigenome and transcriptome (epi-RNA multiome) had been highly demanded because hierarchy and correlation between them can be observed directly through a multi-omics analysis. It particularly requires the method and technique of single-cell owing to their cellular variability. Optimization of demanding methods and techniques for single-cell multi-omics library construction is quite challenging. It requires either the preemptive step of physically separating DNA and RNA molecules from single cells or serial (or dual) enzyme reactions in identical single cells. For example, the single-cell DNA methylome and transcriptome sequencing method (scM&T-seq) physically separated RNA molecules by bead-captured oligo dT primer from DNA of a respective single cell [27]. Single-cell nucleosome, DNA methylation, and transcription sequencing (scNMT-seq), triple multiome also use the same physical compartmentalization of DNA and RNA molecules [28]. Both methods sampled mouse embryonic stem cells and showed links between DNA methylation and transcription, all three molecular layers, and dynamics coupling during differentiation. In particular, multiome analysis of mouse gastrulation utilizing scNMT-seq indicated important change and correlation by the temporal sampling of embryos [71]. Strikingly, mesoderm and endoderm showed global epigenetic change at enhancer regions driven by ten-eleven translocation‒mediated demethylation and concomitant increase of accessibility. In contrast, ectoderm's methylome and global chromatin accessibility are already established in the early epiblast [71]. Furthermore, this study featured regulatory elements associated with different states of primed or remodeled of three primary germ layers with subsequent gene expression profiles in detail, which is an ideal example of what a multiome study is aiming for. Another triple multiome, scChaRM-seq (single-cell chromatin accessibility, RNA barcoding, and DNA methylation sequencing), provided a detailed map of the methylome, chromatin accessibility, and transcriptome in growing human oocytes [29]. They observed a global *de novo* DNA methylation setting that correlates with chromatin accessibility during human oocyte growth. The scTrio-seq (single-cell triple omics sequencing) performed triple multiome of the genome, methylome, and transcriptome and indicated that the copy number variations (CNVs) cause proportional changes in transcription. In contrast, CNVs do not affect DNA methylation in the same regions with an individual mammalian cell [30] and subpopulation within human hepatocellular carcinomas. Despite brilliant methodology and outputs of scM&T-seq, scNMT-seq, scChaRM-seq, and scTrio-seq, their cell throughput is limited from tens to hundreds due to the requirement of laborious manual separation of each cell and physical separation of DNA and RNA molecules before each enzyme reaction. Single-cell combinatorial indexing jointly profiles chromatin accessibility, and mRNA (sci-CAR [31]) increased the cell throughput to thousands of levels, and the method aimed at simultaneous RNA- and ATAC-seq. The sci-CAR is the variant method of single-cell combinatorial indexing RNA sequencing [3,72,73]. All sci-named methods use smart combinatorial cell barcoding, enabling millions of cell throughput and making cell compartmentalization unnecessary. The sci-CAR method generated the multiome data of thousands of cells, which was a substantially higher cell throughput than other multiome methods stated above. The interesting feature of the sci-CAR protocol is the serial enzyme treatment with fresh or fixed nuclei. It implies that the different enzymes can be incubated with nuclei serially, and different pools of DNA and RNA molecules are simultaneously utilized to be a final single multiome library. Regarding the data, sci-CAR reconstructed the chromatin accessibility profiles of mouse kidney cell types with the transcriptome. Although sparsity of resulting data, particularly concerning chromatin accessibility of sci-CAR, was still required for improvement, it provided researchers the idea to develop a following multiome that needs multiple enzyme reactions with high cell throughput. Indeed, the parallel analysis of individual cells for RNA expression and DNA accessibility by sequencing (Paired-seq) was developed based on the sci-CAR methodology to increase cell throughput [32]. Interestingly, tagmentation reaction preceded reverse transcription (RT) in the Paired-seq protocol, whereas RT precedes tagmentation in the sci-CAR protocol. In addition, Paired-seq includes three more rounds of combinatorial barcoding than sci-CAR so that the cell throughput increased to millions of cells. Another method of droplet-based single nucleus chromatin accessibility and RNA expression sequencing (SNARE-seq) [33]) utilizes dual capture beads for tagmented DNA and mRNA [12]. Both methods demonstrated the transcriptome and chromatin accessibility of major and rare cell populations and pinpointed lineage-specific accessible sites of rare cells during mouse neurogenesis. Since multiome directly can show the relationship between epigenetic molecules and transcripts, we can construct the map of gene expression profiles with their causal landscape of the genome at a single-cell level. However, only high cell throughput (>1,000) epi-RNA multiome has been developed for chromatin accessibility (Table 2). The high cell throughput epi-RNA multiome for DNA methylation and histone PTMs are also highly demanded to understand comprehensive regulation of gene expression programs.

## Conclusion

The major epigenetic features such as chromatin accessibility, DNA methylation, and histone modifications show clear cross-relationship and provide cellular identity controlling gene expression landscape. Epigenetic features above have to be observed in each cell owing to their strong characteristics of cellular heterogeneity. The development of single-cell genomics has evolved rapidly over the past decade with technological diversity. However, the development of single-cell epigenomics is slower due to the need for appropriate techniques or optimized methods for each epigenetic modification. Recently, optimization and methods for viewing various single-cell epigenetic changes have been developed in multiple ways and suggest relevant ideas for the new method. Alongside the single-cell epigenomic method, the spatial epigenomic method has also been developed recently [74]. These two methods share the same purpose of understanding the complex interrelationships within a single organism, tissue, tumor, and biological system. In addition, the data of two methods merged in the case of the transcriptome in that spatial transcriptome guides where the cell populations are, and single-cell transcriptome adds high resolution of that information at the single-cell level. This merging analysis strategy can be applied equally to the epigenome analysis. Single-cell RNA genomics has identified cell subpopulations within numerous tissues, and single-cell epigenomics will show their regulatory landscape of the genome at the single-cell level. This integrated analysis leads us to answer the question of ‘How are cell or tissue-specific expression patterns or framework specified and maintained with the same genome?’ with critical epigenetic information for cellular differentiation and the occurrence of various diseases.

## Figures and Tables

**Table 1.** List of current methods of single-cell epigenomic library construction

Method name	Individual cell isolation (or barcoding)	Cell throughput	Pros	Cons	Reference
sciMET^M^	Combinatorial indexing	100‒1,000	Pooled cells input, whole-genome coverage	High cost for sequencing	[14]
scRRBS^M^	Manually or FACS	10‒100	Low cost for sequencing	Restricted coverage	[15]
epi-gSCAR^M^	Manually or FACS	10‒100	Free of bisulfite treatment	Restricted coverage	[16]
scXRBS^M^	Manually or FACS	10‒100	Extended genome coverage (than scRRBS)	-	[17]
scDNase-seq^C^	Combinatorial indexing	10‒100	Pooled cells input	-	[18]
scATAC-seq^C^	Automatically by microfluidic device (Fluidigm)	100‒1,000	Pooled cells input	-	[19]
scMNase-seq^C^	Manually or FACS	10‒100			[20]
scCHIP-seq^H^	Automatically by microfluidic droplet chemistry	100‒1,000	Pooled cells input	High loss of input	[21]
scChIC-seq^H^	Manually or FACS	10‒100	High sensitive enzyme (antibody fused MNase)	-	[22]
iACT-seq^H^	Manually or FACS	100‒1,000	High sensitive enzyme (antibody fused Tn5)	-	[23]
scChIL-seq^H^	Manually or FACS	100‒1,000	High sensitive enzyme (antibody fused Tn5)	-	[24]
CoBATCH^H^	Combinatorial indexing	1,000‒10,000	Pooled cells input, High sensitive enzyme (antibody fused Tn5)	-	[25]
scCUT&Tag^H^	Automatically by microfluidic droplet chemistry	1,000‒10,000	Pooled cells input, High sensitive enzyme (antibody fused Tn5)	-	[26]

M DNA methylation;

C chromatin accessibility;

H histone PTMs;

sciMET, single-cell combinatorial indexing for methylation analysis; scRRBS, single-cell reduced-representation bisulfite sequencing; epi-gSCAR, epigenomics and genomics of single cells analyzed by restriction; scXRBS, single-cell extended-representation bisulfite sequencing; scDNase-seq, single-cell DNase-sequencing; scATAC-seq, Single-cell sequencing assay for transposase-accessible chromatin; scMNase-seq, single-cell micrococcal nuclease sequencing; scCHIP-seq, single-cell chromatin immunoprecipitation followed by sequencing; scChIC-seq, single-cell chromatin immunocleavage sequencing; iACT-seq, index multiplexing antibody-guided chromatin tagmentation sequencing; scChIL-seq, single-cell chromatin integration labelling sequencing; CoBATCH, combinatorial barcoding and targeted chromatin release; scCUT&Tag, single-cell cleavage under targets and tagmentation; FACS, fluorescence-activated cell sorting.

**Table 2.** List of current methods of single-cell multi-omics library construction

Method name	Individual cell isolation (or arcoding)	Cell throughput	Pros	Cons	Reference
scM&T-seq^M, R^	Manually or FACS	10-100	-	Need pre-separation of DNA and RNA	[27]
scNMT-seq^M, C, R^	Manually or FACS	10-100	Triple-omic library	Need pre-separation of DNA and RNA	[28]
scChaRM-seq^M, C, R^	Manually or FACS	10-100	Triple-omic library	Need pre-separation of DNA and RNA	[29]
scTrio-seq^M, CNV, R^	Manually or FACS	10-100	Triple-omic library	Need pre-separation of DNA and RNA	[30]
sci-CAR^C, R^	Combinatorial indexing	1,000-10,000	High throughput multi-omic library	-	[31]
Paired-seq^C, R^	Combinatorial indexing	~1,000,000	High throughput multi-omic library	-	[32]
SNARE-seq^C, R^	Automatically by microfluidic droplet chemistry	1,000-10,000	High throughput multi-omic library	-	[33]

M DNA methylation;

C chromatin accessibility;

CNV copy number variation;

R RNA; scM&T-seq, single-cell DNA methylome and transcriptome sequencing method;

scNMT-seq, single-cell nucleosome, DNA methylation, and transcription sequencing; scChaRM-seq, single-cell chromatin accessibility, RNA barcoding, and DNA methylation sequencing; scTrio-seq, single-cell triple omics sequencing; sci-CAR, single-cell combinatorial indexing jointly profiles chromatin accessibility, and mRNA; Paired-seq, parallel analysis of individual cells for RNA expression and DNA accessibility by sequencing; SNARE-seq, single nucleus chromatin accessibility and RNA expression sequencing;FACS, fluorescence-activated cell sorting.
